# Chronic allergic asthma induces T-cell exhaustion and impairs virus clearance in mice

**DOI:** 10.1186/s12931-023-02448-9

**Published:** 2023-06-17

**Authors:** So Yeon Ahn, Jueun Lee, Dong-Ha Lee, Thi Len Ho, Chau Thuy Tien Le, Eun-Ju Ko

**Affiliations:** 1 Veterinary Medical Research Institute, Jeju National University, 63243 Jeju, Republic of Korea; 2 Department of Veterinary Medicine, College of Veterinary Medicine, Jeju National University, 63243 Jeju, Republic of Korea; 3 Interdisciplinary Graduate Program in Advanced Convergence Technology & Science, Jeju National University, 63243 Jeju, Republic of Korea; 4 Lee Kong Chian School of Medicine, Nanyang Technological University, 639798 Singapore, Singapore; 5 Center for Inflammation, Immunity & Infection, Institute for Biomedical Sciences, Georgia State University, 30303 Atlanta, USA

**Keywords:** Allergic asthma, T-cell exhaustion, Ovalbumin, Influenza virus infection

## Abstract

**Background:**

Allergic asthma, one of the most common types of asthma, is thought to be highly susceptible to respiratory viral infections; however, its pathological mechanism needs to be elucidated. Recent studies have found impaired T-cell function in asthmatic mice. Therefore, we aimed to investigate the way by which asthma induction affects T-cell exhaustion in the lungs and assess the relationship between T-cell exhaustion and influenza viral infection.

**Methods:**

Chronic allergic asthma mice were induced by intranasal injection of ovalbumin for 6 weeks and asthmatic features and T cell populations in lung or airway were assessed. To determine the influenza virus susceptibility, control and asthma mice were challenged with the human influenza virus strain A/Puerto Rico/8/1934 H1N1 and evaluated the survival rate, lung damage, and virus titer.

**Results:**

Six weeks of OVA sensitization and challenge successfully induced chronic allergic asthma in a mouse model showing significant increase of sera IgE level and broncho-pathological features. A significant decrease in interferon-γ-producing T-cell populations and an increase in exhausted T-cell populations in the lungs of OVA-induced asthmatic mice were observed. Asthmatic mice were more susceptible to influenza virus infection than control mice showing lower survival rate and higher virus titer in lung, and a positive correlation existed between T-cell exhaustion in the lung and virus titer.

**Conclusions:**

Asthma induction in mice results in the exhaustion of T-cell immunity, which may contribute to the defective capacity of viral protection. This study demonstrates a correlation between asthma conditions and viral susceptibility by investigating the functional characteristics of T-cells in asthma. Our results provide insights into the development of strategies to overcome the dangers of respiratory viral disease in patients with asthma.

**Supplementary Information:**

The online version contains supplementary material available at 10.1186/s12931-023-02448-9.

## Background

Asthma is a chronic inflammatory airway disease caused by various factors including allergen exposure, chronic viral or bacterial infections, and air pollutants [1]. Asthma is a major public health concern affecting 300 million people worldwide [2]. Allergic asthma is one of the most common types of asthma and is characterized by allergen-specific IgE production, airway hypersensitivity, increased mucous production, eosinophil-dominant T helper type 2 (Th2)-biased inflammation, and structural remodeling of the airway [1, 3].

Patients with asthma are considered to be highly susceptible to respiratory viral infections, such as influenza virus[4, 5]. The commonly discussed possible underlying mechanisms of increased susceptibility of the influenza virus infection in allergic asthma mice are deficient Th-1 antiviral immunity [6] and infection triggered asthma exacerbation. They are categorized as a high-risk group of influenza virus infection, and annual vaccination is recommended for them [7, 8].To understand the mechanisms of disease progression and develop prevention and treatment strategies, an artificial asthma mouse model has been used for in vivo asthma studies since the 1990s [9–11]. Some studies have attempted to determine the relationship between viral infection and asthmatic conditions using an asthmatic mouse model. One reported that influenza virus infection exacerbated asthmatic conditions but not worsen the virus replication[12], and others reported that asthma mice were even more resistant in the early stage of viral infection [13, 14]. Because asthma is a complex multifactorial disease, it is not easy to duplicate every feature of chronic human asthma in a mouse. Moreover, many different factors such as a type of allergens, period of induction, and the condition of virus infection, can impact the results of the asthma condition. It makes diverse views and controversy whether asthma mice are more susceptible to influenza virus infection like patients with asthma.

The immunological conditions of asthma, such as persistent foreign allergen stimulation, can make asthma prone to immunosenescence and lead to exhaustion of T-cells, such as in aging and other chronic immune-mediated inflammatory diseases [15, 16]. Despite the importance of appropriate T cell function in viral protection, little is known about T cell exhaustion in asthmatic condition and the effect to influenza virus infection.

In this study, we investigated the relationship between chronic allergic asthma and influenza virus susceptibility using ovalbumin (OVA)-induced asthma mice model. Also, we tried to elucidate the mechanism in the context of T cell-immunity. To the best of our knowledge, this is the first study demonstrating the relationship between T cell exhaustion and influenza viral infection in asthma mice.

## Methods

### Mice

Six-week-old female BALB/c mice were purchased from Orient Bio and maintained at Jeju National University Animal Facility. All mouse experiments were performed in accordance with the guidelines of Jeju National University approved by the Institutional Animal Care and Use Committee (protocol number 2021-0051). For immunization and infection, mice were anesthetized using isoflurane.

### OVA-induced allergic asthma mouse model

**Fig. 1:**
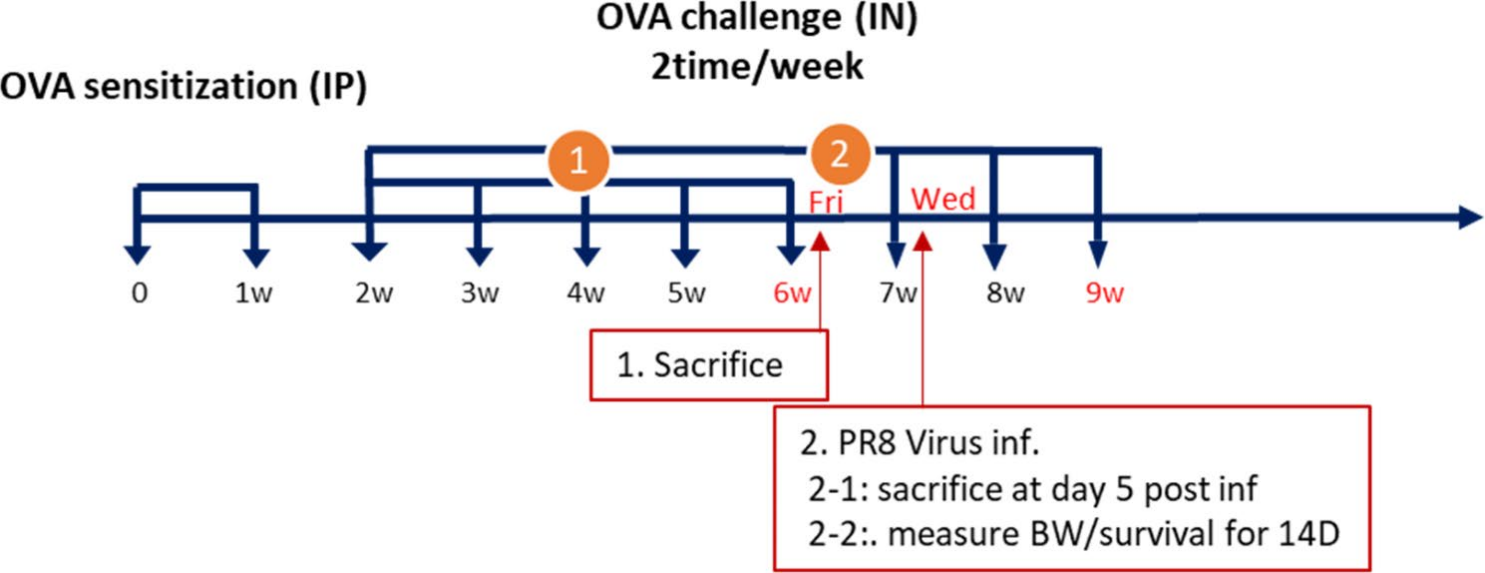
Experimental schedule of OVA sensitization and immunization in asthmatic mice

BALB/c mice (n = 4/group) were sensitized by intraperitoneal injection of 50 µg OVA (Sigma-Aldrich, St. Louis, MO, USA) and 1 mg aluminum hydroxide (InvivoGen) in 200 µL of phosphate-buffered saline (PBS) at weekly intervals for 2 weeks. One week after the second sensitization, mice were challenged intranasally with 50 µg OVA in 50 µL PBS two times per week. The control mice were sensitized and challenged with PBS only. Asthmatic mice were sacrificed 6 weeks after OVA sensitization, and their asthmatic features were evaluated. The sensitization and challenge schedules are shown in Fig. 1A. The experiments were conducted 3-times independently and the representative data were shown. As an indicator of allergic response, the levels of immunoglobulin (Ig) E in sera and the population of eosinophils in the lung and bronchoalveolar lavage fluid (BALF) were measured. The population of inflammatory cells in the lung and BALF, levels of inflammatory cytokines and chemokines, and hematoxylin and eosin (H&E)-stained histological features of lung tissues were analyzed to evaluate lung inflammation. In addition, to investigate the properties of T-cells in asthmatic mice, the Th2/Th1 cell population and the expression of the T-cell exhaustion marker, thymocyte selection-associated HMG BOX (TOX) and programmed cell death receptor-1 (PD-1) were determined using flow cytometry.

### Influenza virus challenge to the asthma mouse model

Human influenza virus strain A/Puerto Rico/8/1934 H1N1 (A/PR8) was obtained from ATCC (VR-95™) and amplified in 9-day old embryonated chicken eggs. The virus was harvested from allantoic fluid and maintained at − 80 °C until use. Mice were divided into four groups (n = 8 each): control, asthma, naïve-infection, and asthma-infection groups. The control and asthma groups were intranasally injected with 50 µL PBS. For the naïve-infection and asthma-infection groups, control or asthma-induced mice, respectively, were intranasally challenged with A/PR8 virus at a dose of 50% lethal dose (LD_50_) 7 weeks after OVA sensitization. The LD50 of the PR8 virus stock was determined using preliminary mouse viral challenge experiments. Mice were sacrificed at two different time points: 5 d after viral infection to evaluate the pathogenesis of influenza and 14 d after infection to monitor the daily body weight change and survival. Viral titers were determined as the 50% egg infectious dose (EID_50_)/mL.

### Sample collection and preparation

Control and asthmatic mice were sacrificed 6 weeks after OVA sensitization, and sera were harvested via centrifugation of blood collected from the caudal vena cava. BALF samples were collected by delivering 1.2 mL PBS through the trachea using a 25-gauge catheter. Lung tissues were obtained separately for histological and cellular/cytokine analyses. For histological analysis, lung tissues were immediately fixed with 10% formalin. Lung tissues for cellular/chemical analysis were mechanically mashed and filtered using a 100-µm cell strainer. We have comparative experiment data of mechanical disassociation method and mechanical and enzymatic method to evaluate loss of T cell populations depending on the methods (Supplementary Fig. 1) Following centrifugation, supernatants were stored at − 80 °C until cytokine enzyme-linked immunosorbent assay (ELISA). Following red blood cell lysis, lung cell pellets were resuspended in 1 mL PBS containing 2% fetal bovine serum (FBS, FACS buffer) for flow cytometry.

Influenza virus challenge groups (naïve, naïve-infection, asthma, and asthma-infection groups) were sacrificed on day 5 post-challenge, and BALF and lung samples were collected and stored in the same way. Lung extracts for virus titration were stored at − 80 ℃ until use.

### Serum OVA-specific IgE ELISA

To measure OVA-specific IgE levels in the serum, the ELISA plate was coated with OVA protein (400 ng/well) before adding serum. Serially diluted sera were added to OVA-coated ELISA plates (400 ng/well) after blocking. A horseradish peroxidase (HRP)-labeled anti-mouse IgE secondary antibody (Southern Biotech) was used to detect antigen-specific IgE in the serum. A tetramethylbenzidine solution was used as the substrate, and the reaction was stopped using sulfuric acid. The optical density was measured at 450 nm.

### Lung virus titration

The lung extracts from influenza-infected mice groups were serially diluted in PBS from 10^− 5^ to 10^− 10^, and 300 µL of each dilution was inoculated into 9-d old embryonated chicken eggs (three eggs per dilution). After incubation for 3 d, 50 µL allantoic fluid was collected and added to a 96-well U-bottom plate. Chicken red blood cells (50 µL, 0.5%) were added to all the wells and incubated for 30 min at room temperature. The 50% endpoint for hemagglutinin activity in chicken RBCs was determined according to the method of Reed and Muench [17].

### Cytokine and chemokine ELISA

Cytokine levels in the BALF and lung extracts were measured using tumor necrosis factor (TNF)-α, interleukin (IL)-6, IL-12 p40 Mouse Uncoated ELISA Kit (Invitrogen), and interferon (IFN)- γ, IL-4, IL-13, C-C motif chemokine ligand 2 (CCL2) DuoSet ELISA kit (R&D system) according to the manufacturers’ protocol.

### Flow cytometry

For cell phenotype staining, harvested cells were blocked with an anti-CD16/32 (clone 2.4G2) antibody after washing with 2% FBS containing PBS (FACS buffer). Each antibody cocktail was subsequently added to the cells and incubated for 30 min at room temperature in the dark. Intracellular cytokine staining for Tox, IL-4, IL-13, and IFN-γ was performed using a BD Cytofix/Cytoperm kit. The data were acquired using the BD FACS DIVA program and analyzed with FlowJo software.

To investigate the inflammatory cell population, the lung and BAL cells were stained with an antibody cocktail containing anti-mouse CD45 (clone 30-F11), CD11b (clone M1/70), CD11c (clone N418), F4/80 (clone BM8), Ly6c (clone AL-21), MHC class II (clone I-A/I-E), CD170 (clone S17007L), and live/dead aqua (L/D). For memory T-cell staining, CD45 (clone 30-F11), CD3 (clone 17A2), CD4 (clone RM4.5), CD8a (clone 53 − 6.7), CD44 (clone IM7), CD62L (clone MEL-14), and L/D were stained. For T-cell exhaustion marker staining, antibodies against CD45 (clone 30-F11), CD3 (clone 17A2), CD4 (clone RM4.5), CD8a (clone 53 − 6.7), PD-1 (clone 29 F.1A12), and TOX (clone TXRX10) were used. The intracellular cytokines IL-4 (clone 11B11) and IFN-γ (clone XMG1.2) in T-cells were stained with CD45 (clone 30-F11), CD3 (clone 17A2), CD4 (clone RM4.5), and CD8a (clone 53 − 6.7) to distinguish Th1/Th2 cells. The gating strategies are shown in Supplementary Fig. 2. The inflammatory cell populations were double-confirmed by FSC/SSC back-gating (Supplementary Fig. 3).

### Lung histology and inflammation scoring

Portions of left lower lobe lung tissue were fixed in 10% neutralized buffered formalin, processed, and embedded in paraffin. Sections were cut to a thickness of 1 μm and stained with H&E. Lung sections were scored from 0 to 4 blindly for peribronchial infiltrates and alveolar involvement using an adapted histological scoring system (Table 1) that was originally described by Dubin et al. [18].

**Table 1:** Lung inflammation scoring of asthma and control mice after influenza infection

Score	Bronchiolar Infiltrate		Alveolar Involvement
	Intraluminal Infiltrate	Peribronchial Infiltrate	
0	None	None	None
1	≦ 25% of visualized lumens; predominate mononuclear	Infiltrate ≦ 4 cells thick	Increased cellularity; no evidence of septal thickening
2	25–50% of visualized lumens; mixed mono- and polymorphonuclear	Infiltrate 5–10 cells thick	Increased cellularity; no evidence of septal thickening
3	50–75% of visualized lumens; predominately polymorphonuclear	25–50% of visualized lumens	Significant celluarity, thickening; edema or blood, obliteration of < 25% of visualized alveolar space
4	Diffuse; predominately polymorphonuclear	Diffuse	Obliteration of > 25% of the alveolar space

### Statistical analysis

All results are presented as mean ± standard error of the mean, and statistical significance was analyzed using GraphPad Prism software 9.2.0 (GraphPad Software Inc.). Significant differences between two groups were analyzed using unpaired t-test. One-way ANOVA or two-way ANOVA was used for the comparison of multiple groups. Statistical significance was set at p < 0.05.

## Results

### Six weeks of OVA sensitization and challenge successfully induces chronic allergic asthma in a mouse model

**Fig. 2:**
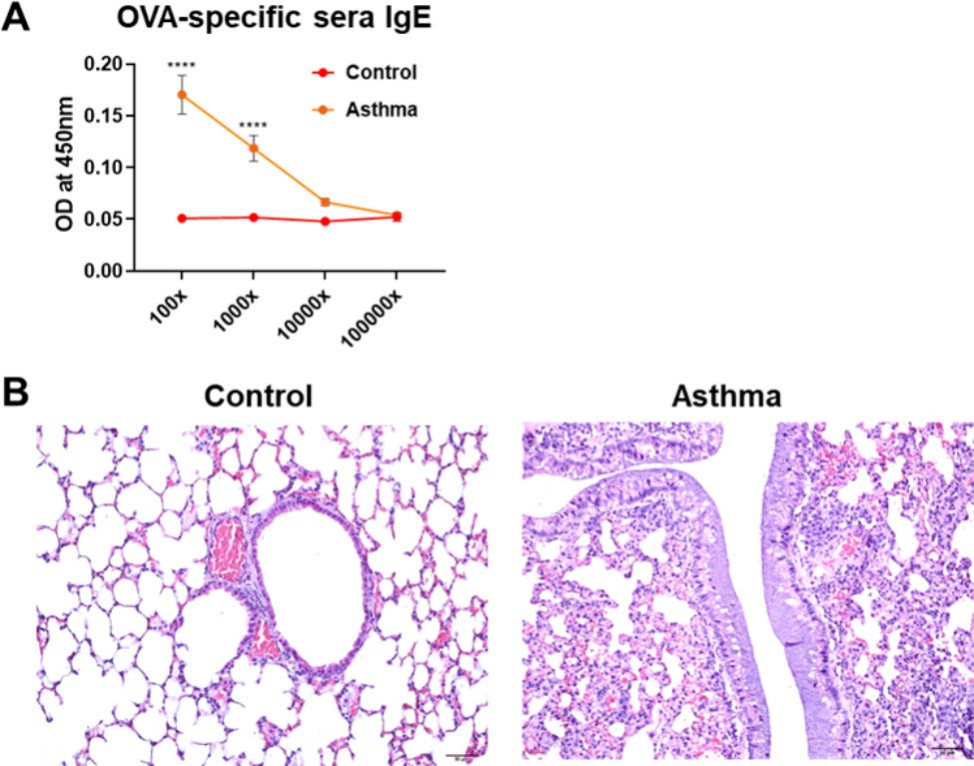
**OVA sensitization and challenge induced chronic asthma in mice. Sera and lung Samples were collected at 6 weeks post OVA sensitization.** (**A**) OVA-specific IgE level in sera were measured by ELISA. Data of OVA-specific sera IgE were shown in mean ± SEM. For statistical analysis, two-way ANOVA and Sidak’s-multiple comparison test were performed. ****p < 0.0001 between the indicated groups (n = 4/group). (**B**) Lung tissues were stained with hematoxylin and eosin (H&E) and the representative histological analysis of each group at magnification x200 was shown

**Fig. 3:**
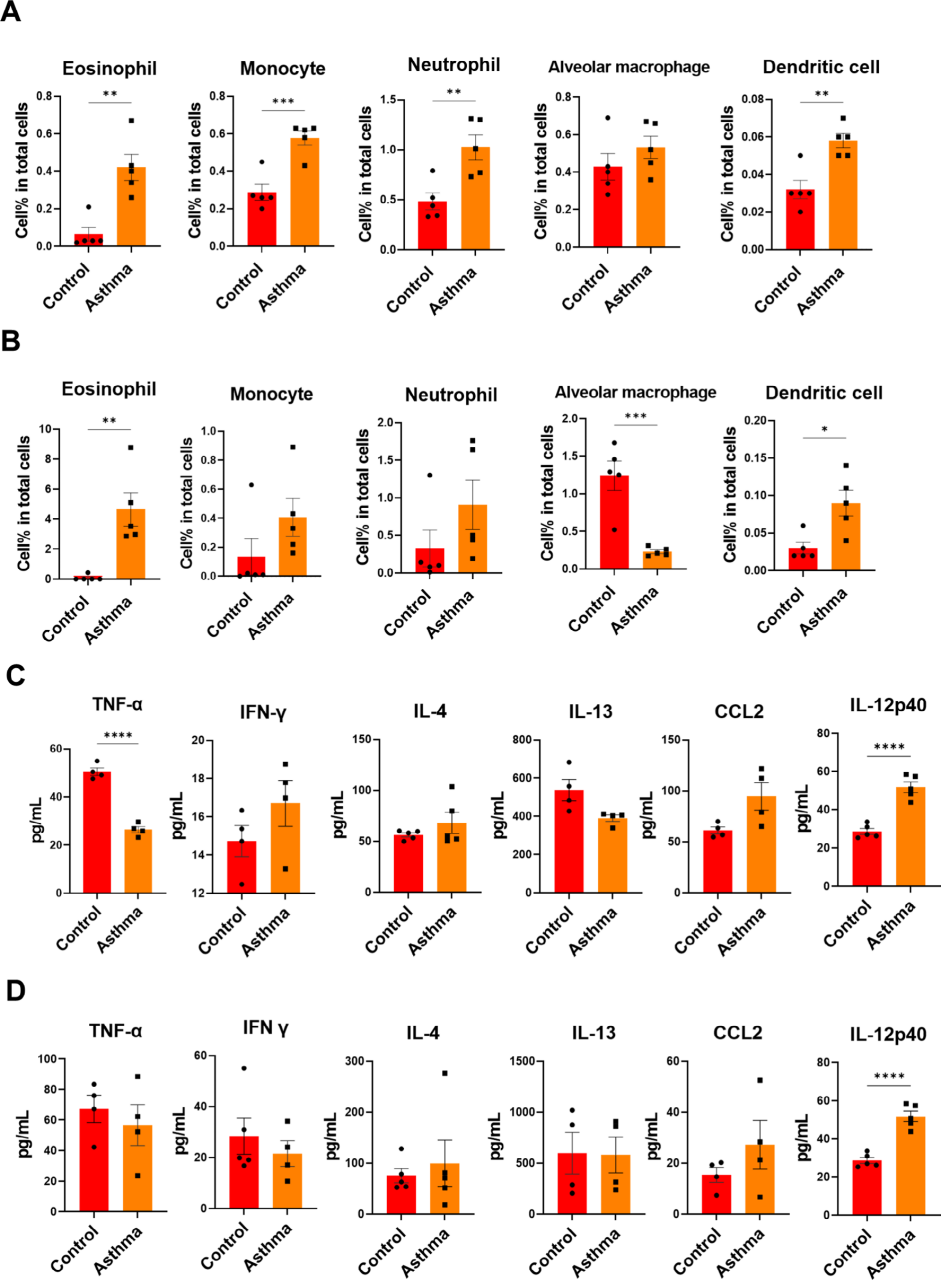
**Inflammatory response in lung and BALF after asthma induction. Samples were collected at 6 weeks post OVA sensitization.** Inflammatory cell population in Lung (**A**) and BAL (**B**) were measured by flow cytometry. Inflammatory cytokine and chemokine level in Lung (**C**) and BAL (**D**) were measured by ELISA. All data were shown in mean ± SEM. For statistical analysis, unpaired t test was performed. *p < 0.05; **p < 0.01; ***p < 0.001 ****p < 0.0001 between the indicated groups (n = 4/group)

To establish a chronic asthma mouse model, mice were sensitized intraperitoneally with OVA plus alum for 2 weeks and subsequently challenged intranasally with OVA weekly. To evaluate the asthmatic features of the mouse model, mice were sacrificed 6 weeks after OVA sensitization. Asthmatic mice showed a significantly increased serum level of OVA-specific IgE compared to control mice (Fig. 2A). Representative broncho-pathological characteristics of chronic asthma, such as infiltration of inflammatory cells in the bronchial region, epithelial detachment, airway thickening, and mucous plugging, were observed in the lungs of OVA-induced asthmatic mice (Fig. 2B). Flow cytometry analysis revealed inflammatory cell infiltration including eosinophil, monocyte, neutrophil and dendritic cells (DCs) in the lungs and BALF of asthmatic mice (Fig. 3A, B and supplementary Fig. 4). Regarding inflammatory cytokines, we observed an increase in the levels of CCL2 and IL-12p40 in asthmatic mice compared to those in control mice; however, only the increased level of IL-12p40 was significant (Fig. 3C, D). TNF-α level was significantly decreased in the lungs of asthmatic mice than in those of control mice (Fig. 3C). IL-4 level marginally increased in asthmatic mice; however, the difference was not statistically significant (Fig. 3C, D). In addition, there was no difference in the levels of IFN-γ and IL-13 between the control and asthmatic mice. These results indicate that the 6-week OVA-sensitization and challenge protocol successfully induced chronic allergic inflammation and structural changes in the airway of mice, despite marginal changes in the levels of inflammatory cytokines.

### Asthmatic mice show Th2-biased T-cell populations with increased exhausted T-cells

**Fig. 4:**
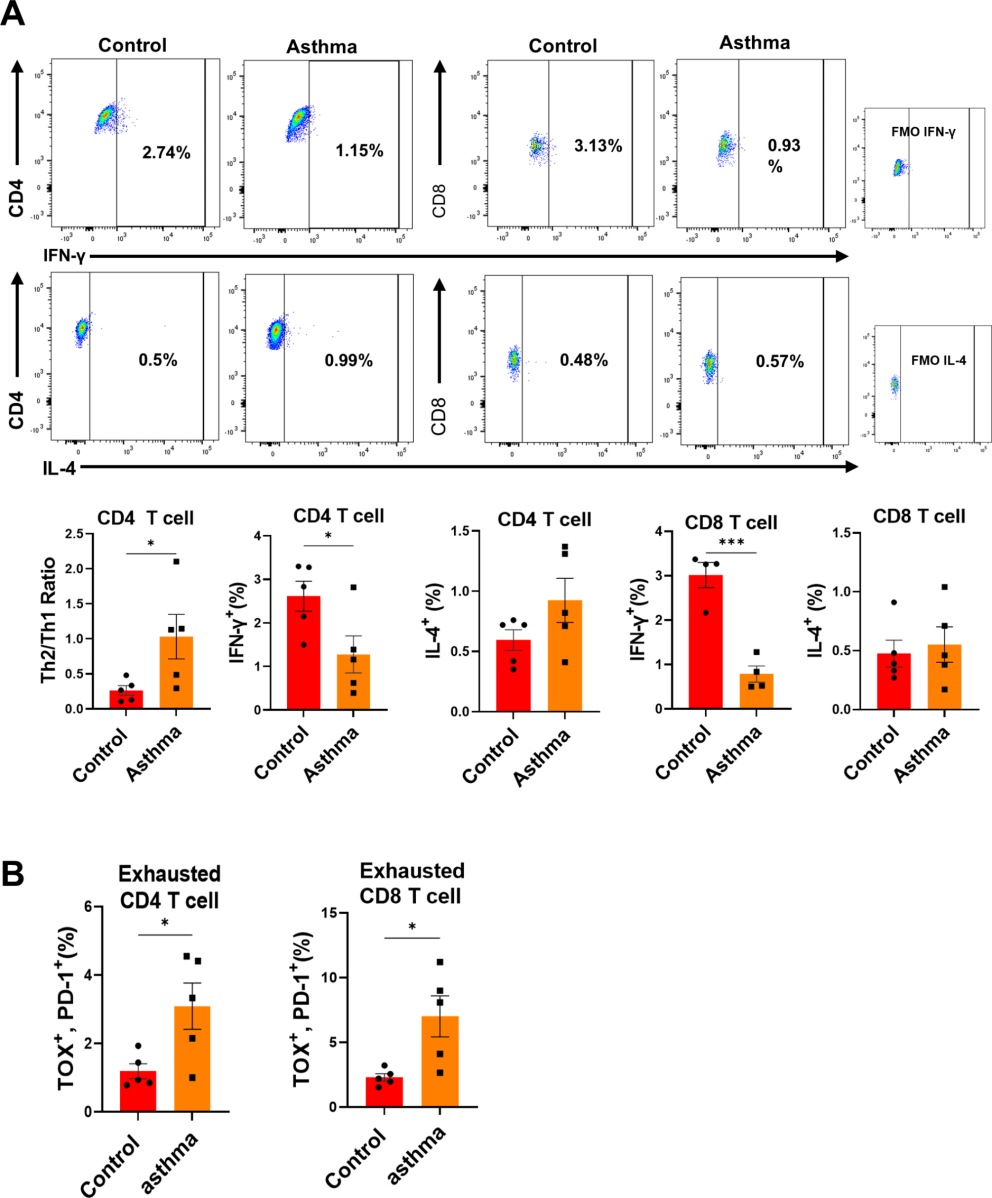
**Th2/Th1 ratio and T cell exhaustion marker expression of lung cells of asthma mice.** (**A**) Intracellular cytokine level of IL-4 and IFN-γ in T cell of the lung of control and asthma mice were measured. (**B**) T cell exhaustion marker expression in CD4 and CD8 T cells in lung. Data were measured by flow cytometry. All result were shown in mean ± SEM. For statistical analysis, unpaired T tests were performed. *p < 0.05, **p < 0.01, ***p < 0.001 between the indicated groups (n = 4/group)

To further investigate the T-cell condition of asthmatic mice, we assessed the Th2- and Th1-biased cell populations of CD4 T-cells in the lung by measuring the intracellular levels of IL-4 and IFN-γ, respectively. The ratio of Th2/Th1 cells to CD4 T-cells significantly increased, indicating that OVA-induced asthma elicited Th2-biased T-cell differentiation. The IFN-γ ^+^ CD8 T-cell population was lower in asthmatic mice than in control mice (Fig. 4A). The populations of PD-1^+^TOX^+^ CD4 and CD8 T-cells were significantly higher in the lungs of asthmatic mice than in the lungs of control mice (Fig. 4B). PD-1 and TOX are well-known T-cell exhaustion markers [19]. These data suggested that chronic asthma conditions resulting from a long and persistent stimulation by the foreign antigen could make T-cells over-exhausted, resulting in loss of their cytokine production function.

### Chronic asthma increases susceptibility to PR8 influenza virus infection

**Fig. 5:**
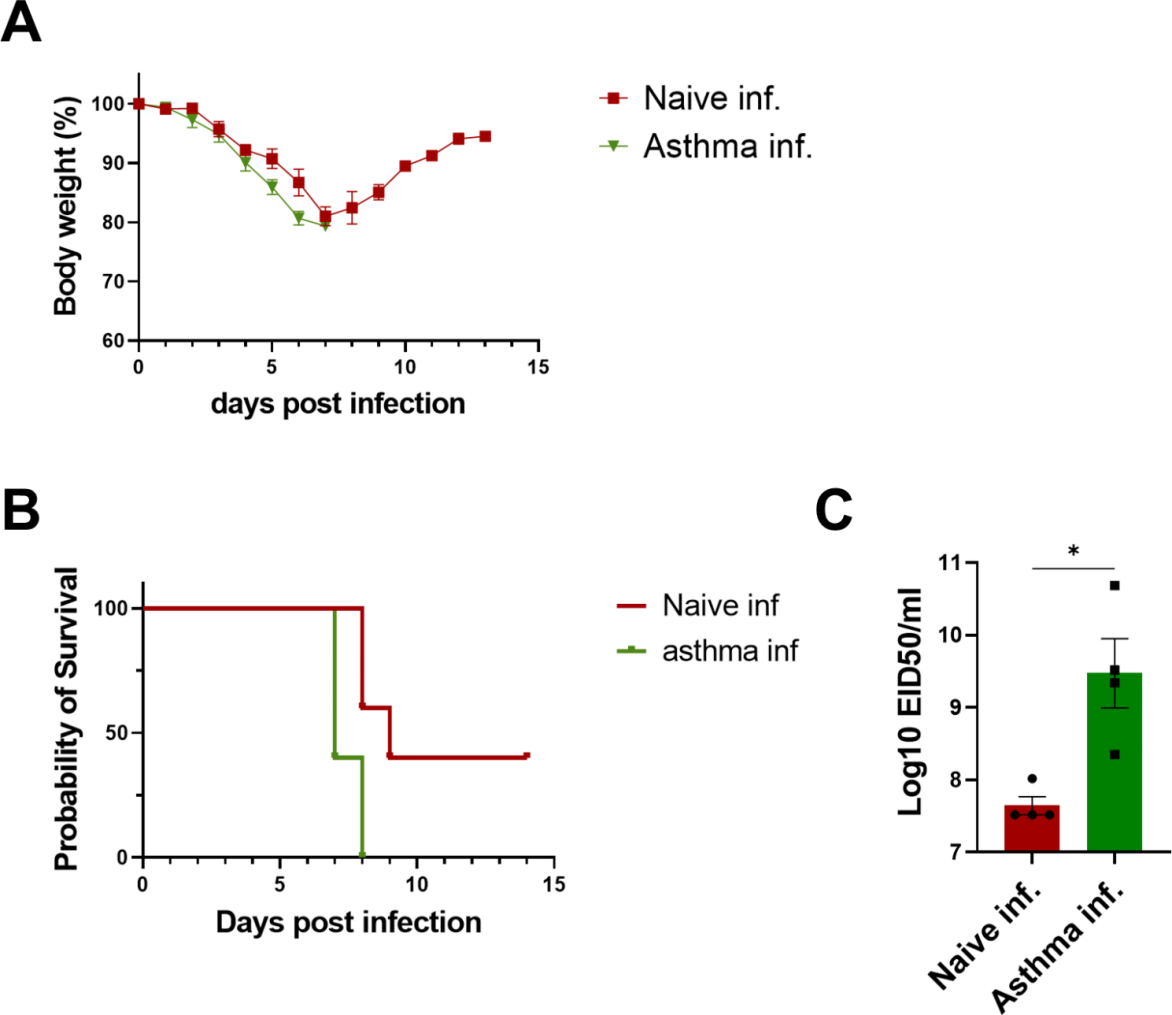
**Influenza virus susceptibility and clearance of asthma mice. (A)** Body weight changes were monitored for 14 days after infection with A/PR8 influenza virus (1xLD_50_). **(B)** Survival rate of the asthma mice after infection with A/PR8 virus. **(C)** Lung viral titers at day 5 post infection. Data of virus titration were shown in mean ± SEM. For statistical analysis, unpaired t test was performed. *p < 0.05 between the Naïve infection group and Asthma infection group (n = 4/group)

To evaluate the effects of chronic asthma on the pathogenesis of viral infection, control and asthmatic mice were challenged with A/PR8 virus at a dose of LD_50_ 7 weeks after OVA sensitization. Both the naïve-infection and asthma-infection groups experienced weight loss; however, the asthma-infection group lost more weight from 5 d post-infection (Fig. 5A). The asthma-infection group exhibited a 0% survival rate, whereas the naïve-infection group showed a 50% survival rate (Fig. 5B). In addition, the virus titer was significantly higher in the asthma-infection group than in the naïve-infection group 5 d after viral challenge (Fig. 5C).

**Fig. 6:**
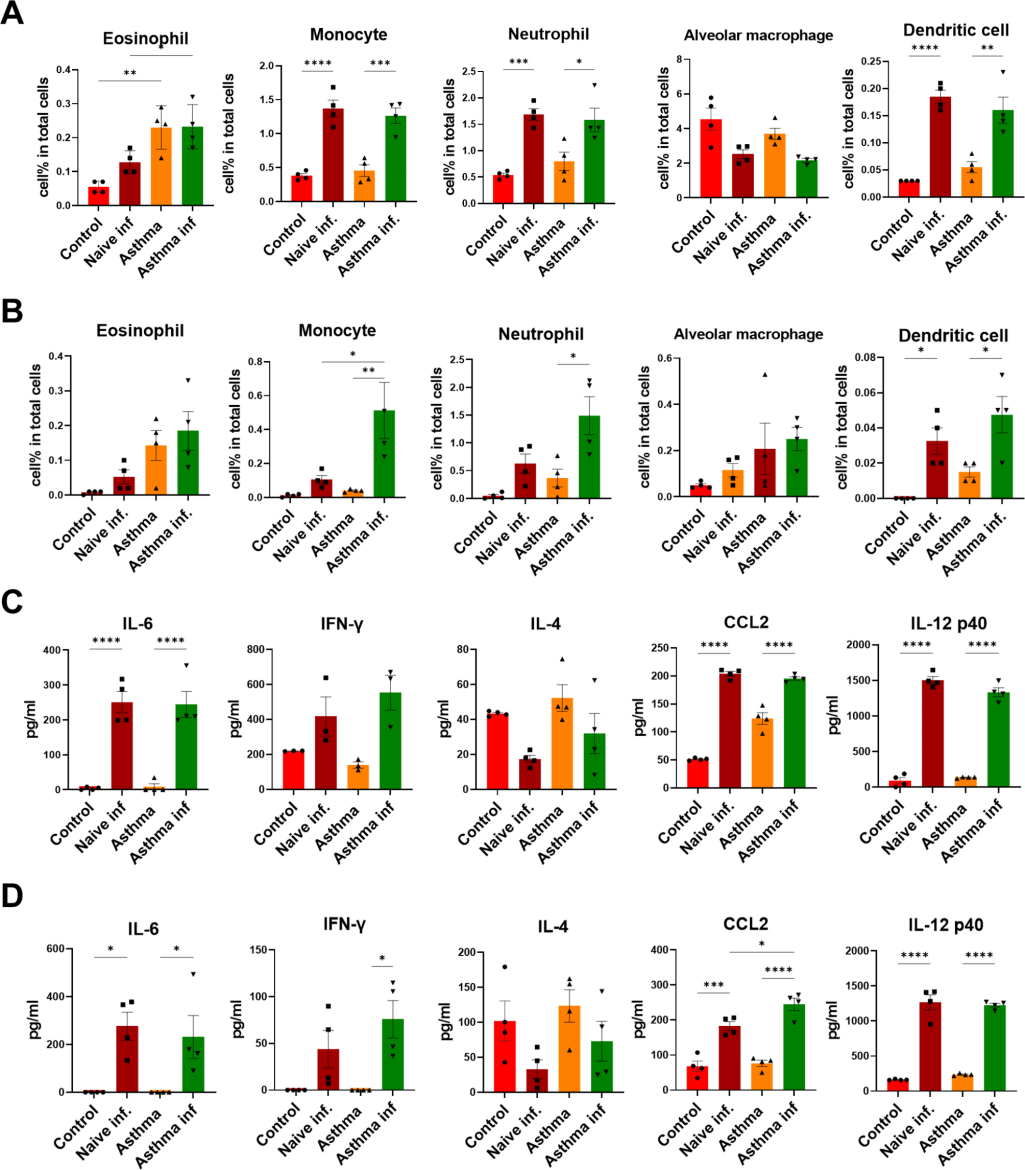
**Airway inflammation was increased after PR8 influenza virus infection. Samples were collected at 5 days post infection.** Inflammatory cell population in Lung (**A**) and BAL (**B**) were measured by flow cytometry. Inflammatory cytokine and chemokine levels in Lung (**C**) and BAL (**D**) were measured by ELISA. All data were shown in mean ± SEM. For statistical analysis, one-way ANOVA and Tukey’s post-multiple comparison test were performed. *p < 0.05; **p < 0.01; ***p < 0.001 ****p < 0.0001 between the indicated groups (n = 4/group)

Inflammation in the airway after A/PR8 influenza infection was determined 5 d post-challenge. The inflammatory cell population of the lung and BAL and the inflammation score of lung tissues were assessed using flow cytometry and H&E staining, respectively. The eosinophil counts were similarly high in the lungs of the asthma and asthma-infection groups following A/PR8 challenge. The counts of other inflammatory cells, such as monocytes, neutrophils, and DC, increased after the challenge in both the naïve-infection and asthma-infection groups (Fig. 6A). BAL cell populations maintained the patterns of lung cell populations; however, the asthma-infection group showed the highest percentage of all inflammatory cell populations (Fig. 6B). Although there was no drastic difference in the inflammatory cell populations in the lung, except for eosinophils, between the naïve-infection and asthma-infection groups, the asthma-infection group exhibited significantly increased inflammatory cell counts in BAL. The cytokine production of IL-6, CCL-2, and IL-12p40 in the lungs and BAL significantly increased after viral challenge in both the naïve-infection and asthma-infection groups. The production of IFN-γ also increased post-challenge; however, IL-4 levels decreased after viral challenge (Fig. 6C, D).

**Fig. 7:**
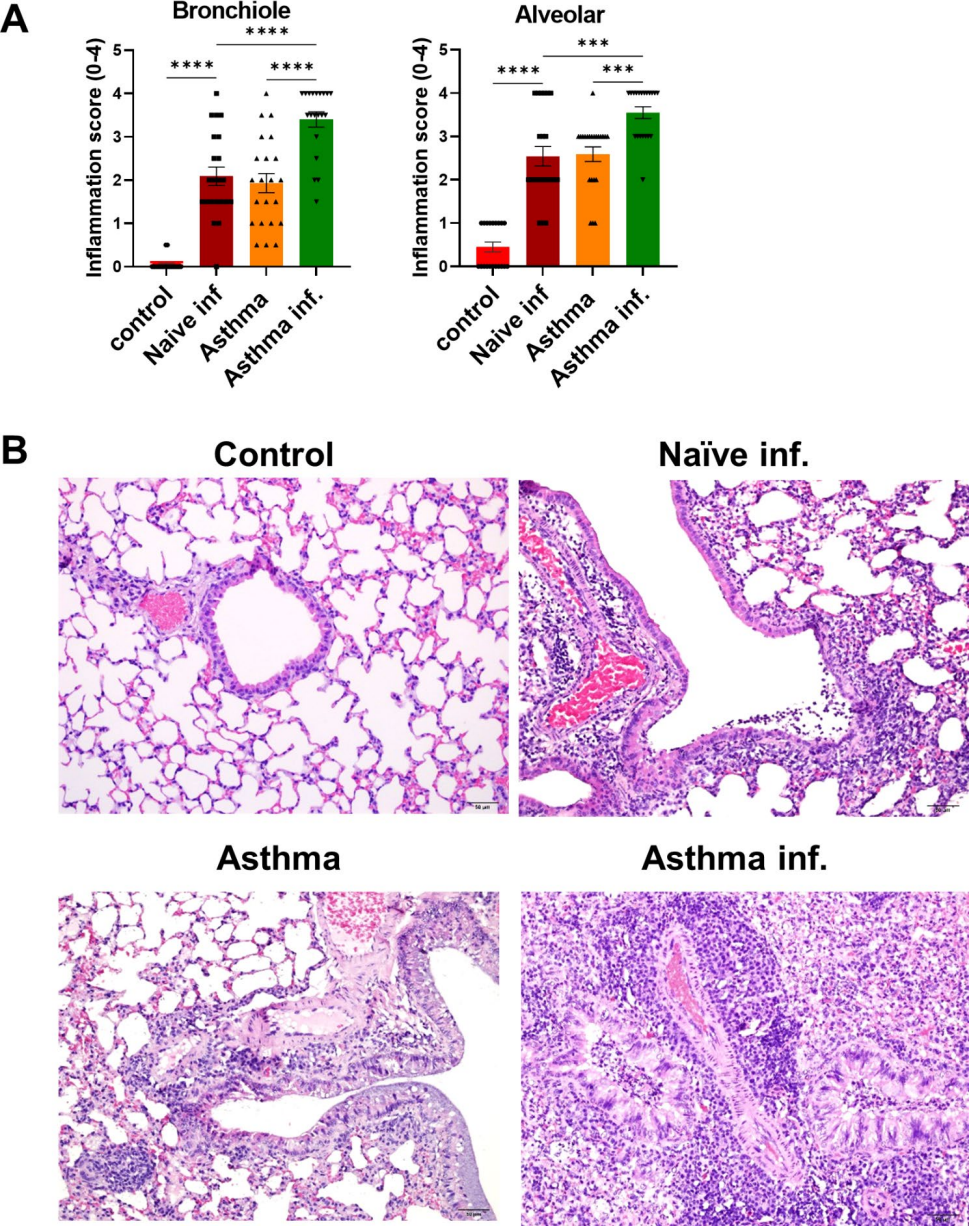
**Lung histological analysis and scoring of control and asthma mice after A/PR8 influenza infection.** The inflammation score in lung (**A**) and the representative histology pictures of lung tissues at magnification x200 (**B**) after A/PR8 influenza virus infection. Lung tissues were collected at 5 days post infection and stained with hematoxylin and eosin (H&E). Lung inflammation score were blindly quantified (20–22 pictures/group) between 0–3, using publishing criteria: 0 (normal), 1 (mild), 2 (moderate) and 3 (severe). All data were shown in mean ± SEM. For statistical analysis, one-way ANOVA and Tukey’s post-multiple comparison test were performed. *p < 0.05; **p < 0.01; ***p < 0.001 ****p < 0.0001 between the indicated groups

Histopathological analysis of the lungs showed that the asthma-infection group had significantly higher inflammatory scores than the naïve-infection and asthma groups in both bronchiole and alveolar features (Fig. 7A). Representative histopathological images of the lung are shown in Fig. 7B.

Collectively, our results showed that asthmatic mice were more susceptible to PR8 influenza virus infection with an accelerated viral replication. Asthmatic conditions also appeared to elicit more severe inflammation at the infection site during viral infection.

### T-cell exhaustion in asthmatic mice is aggravated after influenza virus infection and correlates with viral titers

**Fig. 8:**
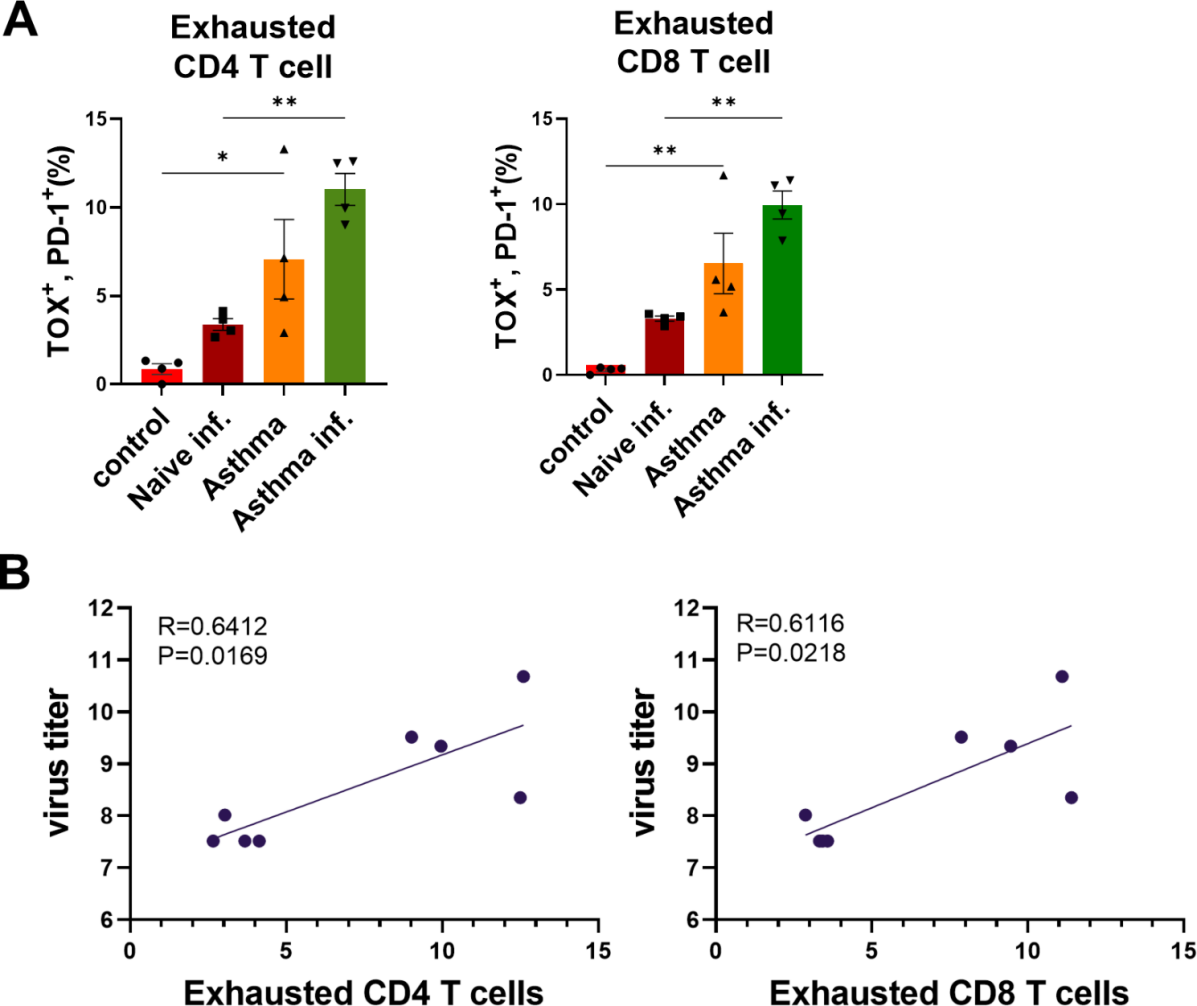
**T cell exhaustion marker expression of lung cells after PR8 influenza virus infection and correlation between the percentage of exhausted T cells and virus titer in mice. (A)** Lung samples were collected at 5 days post infection and T cell exhaustion marker expression in CD4 and CD8 T cells were measured by flow cytometry. **(B)** The correlation analysis between the virus titer and percentage of exhausted CD4, CD8 T cell in influenza infected mice. Data of T cell exhaustion were shown in mean ± SEM. For statistical analysis, one-way ANOVA and Tukey’s post-multiple comparison test were performed. *p < 0.05; **p < 0.01 between the indicated groups (n = 4/group). The correlations between the virus titer and the percentage of exhausted T cells were analyzed using the Person’s correlation analysis. p < 0.05 was considered statistically significant

T-cell exhaustion following PR8 viral infection was determined and compared between groups. The percentages of exhausted CD4 and CD8 T-cells in the naïve-infection group were marginally higher than those in the control group, yet lower than those in the asthma group. T-cell exhaustion was higher in the asthma-infection group than in the asthma group and more than twice as high as that in the naïve-infection group (Fig. 8A). This result suggested that T-cells in chronic asthmatic mice have already been under considerable exhausted conditions and that the condition can be worsened by virus invasion.

We also analyzed the correlation between exhausted T-cells and virus titers in mice following influenza virus infection and found a positive correlation between the percentage of exhausted CD4 and CD8 T-cells and the virus titer (Fig. 8B).

## Discussion

The immunization protocol for OVA allergen exposure in this study was modified based on several protocols showing key characteristics of chronic asthma, including as airway remodeling with persistent airway hyper-responsiveness [20, 21]. Our 6-week OVA-sensitized and virus-challenged mice successfully developed chronic allergic inflammation and structural changes in their airways. In addition, persistent stimulation of allergens induced exhaustion of CD4 and CD8 T-cells in the lungs of asthmatic mice. There was no significant increase in the levels of cytokines in the lungs, except for that of IL-12p40. We could assume based on the current knowledge, that the Th-2 cytokine response was strongly increased in acute period of asthma, up to 18–25 days of immunization [22–24], and got weaker in chronic period. Chronic period of asthma rather showed heterogenic response including increased level of IFN-gamma and IL-17 [25, 26]. IL-23 cytokine and Th-17cells plays pathogenic roles in chronic inflammation disease including many autoimmune diseases [27]. Several studies suggests that these cytokines are even involved in pathogenesis of chronic asthma mice model [28]. Therefore, the increase of IL-12p40 could be a result of the increase of IL-23, like autoimmune disease, regarding of that other Type 1 inflammatory cytokine levels were not increased and showing decreased Th-1 T cell population and increase of exhausted T cell population in asthma mice… Other cellular changes were clear, and the ratio of Th2/Th1 CD4 T-cells in the lungs of asthmatic mice significantly increased. Notably, the increase in IL-4^+^ CD4 T-cells was marginal, and the change in the T-cell ratio was potentially because of the considerable decrease in the population of IFN-γ^+^ CD4 T-cells. A significant decrease in the population of IFN-γ^+^ CD8 T-cells was also observed. The loss of cytokine production and high expression of PD-1 and TOX in the T-cells of the lungs implies exhaustion of T-cells in asthmatic mice.

Functional exhaustion of T-cells has been documented during aging, chronic inflammatory diseases, and chronic viral infections such as human immunodeficiency virus, hepatitis B virus, and hepatitis C virus infections. The capacity of the immune system is limited. Sustained stimulation, such as viral antigens, autoantigens, and allergens, causes T-cell dysfunction that results in susceptibility to diseases [29, 30]. The increased exhausted cell population and functional defects of T-cells are acutely connected to illness progression in chronic viral infections, failing to achieve viral clearance [31, 32]. However, most of those studies have focused on how persistent chronic viral infection overwhelms host T-cell function and the relationship to disease pathogenesis. Our influenza viral-challenged asthma mouse model revealed a relationship between pre-existing exhaustion in T-cells and host protection against new viral infection. A/PR8 influenza virus infection aggregated the exhaustion of CD4 and CD8 T-cells, and their percentages were significantly higher in asthma-infected mice than in naïve-infected mice. In addition, they exhibited higher mortality after an LD_50_ of influenza virus infection, showing a 0% survival rate, compared to the control mice. The virus titer significantly increased in asthma conditions and positively correlated with the percentage of exhausted T-cells. A recent study reported dysfunction of effector memory CD8 T cells in the asthma mice, which can be one of evidence of T-cell exhaustion [33]. We believe that the defect of broad T-cell subsets during the progression of exhaustion contributed the failure of viral clearance in asthma condition.

In this study, we demonstrated that 6-week OVA-induced allergic asthma mice can implement the increased influenza virus susceptibility and we suggest the T cell exhaustion as an important feature associated with the susceptibility of influenza virus in chronic allergic asthma mice. However, we could not discriminate the contribution of each factor to mortality: T cell exhaustion or inflammation in airway, virus replication or asthma exacerbation. Therefore, further studies for figuring out those questions are needed to develop a strategy for prevention and alleviating of pathogenesis of influenza virus infection in asthma patients.

## Conclusions

Asthma is an important chronic health condition that increases the risk of respiratory viral infections by aggravating inflammation. Numerous research teams have studied the mechanisms underlying asthma to prevent and alleviate disease exacerbation caused by respiratory viral infections. This study highlights the functional characteristics of T-cells in chronic asthma and their roles in influenza virus infection, suggesting a critical consideration for developing a strategy to overcome the exhaustion of T-cells and reduce the risk of influenza and other viral infections in patients with asthma.

## Electronic supplementary material

Below is the link to the electronic supplementary material.

Supplementary Material 1: Supplementary method Lung disassociation methods

## Data Availability

Data will be made available on request.

## Abbreviations

A/PR8: Human influenza virus strain A/Puerto Rico/8/1934 H1N1
BALF: Bronchoalveolar lavage fluid
EID50: 50% egg infectious dose
ELISA: Enzyme-linked immunosorbent assay
FBS: Fetal bovine serum
H&E: Ematoxylin and eosinh
HRP: Horseradish peroxidase
LD50: 50% lethal dose
OVA: Ovalbumin
PBS: Phosphate-buffered saline
PD-1: Programmed cell death receptor-1
Th1/2: T helper type 1/2
TOX: Thymocyte selection-associated HMG BOX

## References

[1] Kim HY, DeKruyff RH, Umetsu DT (2010). The many paths to asthma: phenotype shaped by innate and adaptive immunity. Nat Immunol.

[2] Akinbami LJ, Moorman JE, Liu X. Asthma prevalence, health care use, and mortality: United States, 2005–2009. Natl Health Stat Report 2011:1–14. 21355352

[3] Cohn L, Elias JA, Chupp GL (2004). Asthma: mechanisms of disease persistence and progression. Annu Rev Immunol.

[4] Fowlkes AL, Arguin P, Biggerstaff MS, Gindler J, Blau D, Jain S, Dhara R, McLaughlin J, Turnipseed E, Meyer JJ (2011). Epidemiology of 2009 pandemic influenza A (H1N1) deaths in the United States, April-July 2009. Clin Infect Dis.

[5] Van Kerkhove MD, Vandemaele KA, Shinde V, Jaramillo-Gutierrez G, Koukounari A, Donnelly CA, Carlino LO, Owen R, Paterson B, Pelletier L (2011). Risk factors for severe outcomes following 2009 influenza A (H1N1) infection: a global pooled analysis. PLoS Med.

[6] Message SD, Laza-Stanca V, Mallia P, Parker HL, Zhu J, Kebadze T, Contoli M, Sanderson G, Kon OM, Papi A (2008). Rhinovirus-induced lower respiratory illness is increased in asthma and related to virus load and Th1/2 cytokine and IL-10 production. Proc Natl Acad Sci U S A.

[7] Kloepfer KM, Olenec JP, Lee WM, Liu G, Vrtis RF, Roberg KA, Evans MD, Gangnon RE, Lemanske RF, Gern JE (2012). Increased H1N1 infection rate in children with asthma. Am J Respir Crit Care Med.

[8] Cates CJ, Rowe BH. Vaccines for preventing influenza in people with asthma. Cochrane Database Syst Rev 2013:CD000364. 10.1002/14651858.CD000364 11034684

[9] Conde E, Bertrand R, Balbino B, Bonnefoy J, Stackowicz J, Caillot N, Colaone F, Hamdi S, Houmadi R, Loste A (2021). Dual vaccination against IL-4 and IL-13 protects against chronic allergic asthma in mice. Nat Commun.

[10] McKinley L, Alcorn JF, Peterson A, Dupont RB, Kapadia S, Logar A, Henry A, Irvin CG, Piganelli JD, Ray A, Kolls JK (2008). TH17 cells mediate steroid-resistant airway inflammation and airway hyperresponsiveness in mice. J Immunol.

[11] Henderson WR, Lewis DB, Albert RK, Zhang Y, Lamm WJ, Chiang GK, Jones F, Eriksen P, Tien YT, Jonas M, Chi EY (1996). The importance of leukotrienes in airway inflammation in a mouse model of asthma. J Exp Med.

[12] Kawaguchi A, Suzuki T, Ohara Y, Takahashi K, Sato Y, Ainai A, Nagata N, Tashiro M, Hasegawa H (2017). Impacts of allergic airway inflammation on lung pathology in a mouse model of influenza a virus infection. PLoS ONE.

[13] Ishikawa H, Sasaki H, Fukui T, Fujita K, Kutsukake E, Matsumoto T (2012). Mice with asthma are more resistant to influenza virus infection and NK cells activated by the induction of asthma have potentially protective effects. J Clin Immunol.

[14] An S, Jeon YJ, Jo A, Lim HJ, Han YE, Cho SW, Kim HY, Kim HJ (2018). Initial Influenza Virus Replication can be limited in allergic asthma through Rapid induction of type III interferons in respiratory epithelium. Front Immunol.

[15] Goronzy JJ, Weyand CM (2013). Understanding immunosenescence to improve responses to vaccines. Nat Immunol.

[16] Gao Z, Feng Y, Xu J, Liang J (2022). T-cell exhaustion in immune-mediated inflammatory diseases: new implications for immunotherapy. Front Immunol.

[17] REED LJ, MUENCH H (1938). A SIMPLE METHOD OF ESTIMATING FIFTY PER CENT ENDPOINTS12. Am J Epidemiol.

[18] Dubin PJ, Kolls JK (2007). IL-23 mediates inflammatory responses to mucoid Pseudomonas aeruginosa lung infection in mice. Am J Physiol Lung Cell Mol Physiol.

[19] Khan O, Giles JR, McDonald S, Manne S, Ngiow SF, Patel KP, Werner MT, Huang AC, Alexander KA, Wu JE (2019). TOX transcriptionally and epigenetically programs CD8(+) T cell exhaustion. Nature.

[20] Temelkovski J, Hogan SP, Shepherd DP, Foster PS, Kumar RK (1998). An improved murine model of asthma: selective airway inflammation, epithelial lesions and increased methacholine responsiveness following chronic exposure to aerosolised allergen. Thorax.

[21] Henderson WR, Tang LO, Chu SJ, Tsao SM, Chiang GK, Jones F, Jonas M, Pae C, Wang H, Chi EY (2002). A role for cysteinyl leukotrienes in airway remodeling in a mouse asthma model. Am J Respir Crit Care Med.

[22] Kim DI, Song MK, Lee K (2019). Comparison of asthma phenotypes in OVA-induced mice challenged via inhaled and intranasal routes. BMC Pulm Med.

[23] Dai R, Yu Y, Yan G, Hou X, Ni Y, Shi G (2018). Intratracheal administration of adipose derived mesenchymal stem cells alleviates chronic asthma in a mouse model. BMC Pulm Med.

[24] Yao Y, Zeng QX, Deng XQ, Tang GN, Guo JB, Sun YQ, Ru K, Rizzo AN, Shi JB, Fu QL (2015). Connexin 43 Upregulation in mouse lungs during Ovalbumin-Induced Asthma. PLoS ONE.

[25] Kim MS, Cho KA, Cho YJ, Woo SY (2013). Effects of interleukin-9 blockade on chronic airway inflammation in murine asthma models. Allergy Asthma Immunol Res.

[26] McMillan SJ, Xanthou G, Lloyd CM (2005). Therapeutic administration of Budesonide ameliorates allergen-induced airway remodelling. Clin Exp Allergy.

[27] McGeachy MJ, Cua DJ (2008). Th17 cell differentiation: the long and winding road. Immunity.

[28] Nakajima H, Hirose K (2010). Role of IL-23 and Th17 cells in Airway inflammation in Asthma. Immune Netw.

[29] Mueller SN, Ahmed R (2009). High antigen levels are the cause of T cell exhaustion during chronic viral infection. Proc Natl Acad Sci U S A.

[30] Gavazzi G, Krause KH (2002). Ageing and infection. Lancet Infect Dis.

[31] Fisicaro P, Barili V, Rossi M, Montali I, Vecchi A, Acerbi G, Laccabue D, Zecca A, Penna A, Missale G (2020). Pathogenetic mechanisms of T cell dysfunction in chronic HBV infection and related therapeutic approaches. Front Immunol.

[32] Ye B, Liu X, Li X, Kong H, Tian L, Chen Y (2015). T-cell exhaustion in chronic hepatitis B infection: current knowledge and clinical significance. Cell Death Dis.

[33] Zhang H, Liu S, Li Y, Li J, Ni C, Yang M, Dong J, Wang Z, Qin Z (2022). Dysfunction of S100A4(+) effector memory CD8(+) T cells aggravates asthma. Eur J Immunol.

