# Supplementary material for: Chronic allergic asthma induces T-cell exhaustion and impairs virus clearance in mice
Source: Respir Res. 2023 Jun 17;24:160. doi: 10.1186/s12931-023-02448-9 (PMC10332059; doi:10.1186/s12931-023-02448-9)

Supplementary method – Lung disassociation methods

Eight-week Female Balb/c mice (n=3) were infected with 1×LD50 of influenza virus to induce exhausted T cell populations. Mice were sacrificed at 8 day post infection and lungs were collected. We divided each lungs into 2 samples in 6-well plates, filled with 2 ml of RPMI media. Single cells were obtained mechanically mashed and filtered using a 100-μm cell strainer from one sample (mechanical only). The other one (enaymatic+mechanical) was minced into small pieces and incubated with 2.5 mg/ml Collagenase D and 30 μg/ml DNase I for 1 hour at 37°C as using the modified protocol based on the manufacture's protocol. After the digestion, samples were filtered using a 100-μm cell strainer. Following red blood cell lysis, lung cell pellets were resuspended in 3 mL PBS containing 2% fetal bovine serum (FBS, FACS buffer), and divided in 3 tubes for flow cytometry. Intracellular cytokine staining has performed in both sample in equal condition as described in the manuscript.

Supplementary figure 1

A. Comparisons of total and exhausted T cell populations depending on the lung disassociation methods

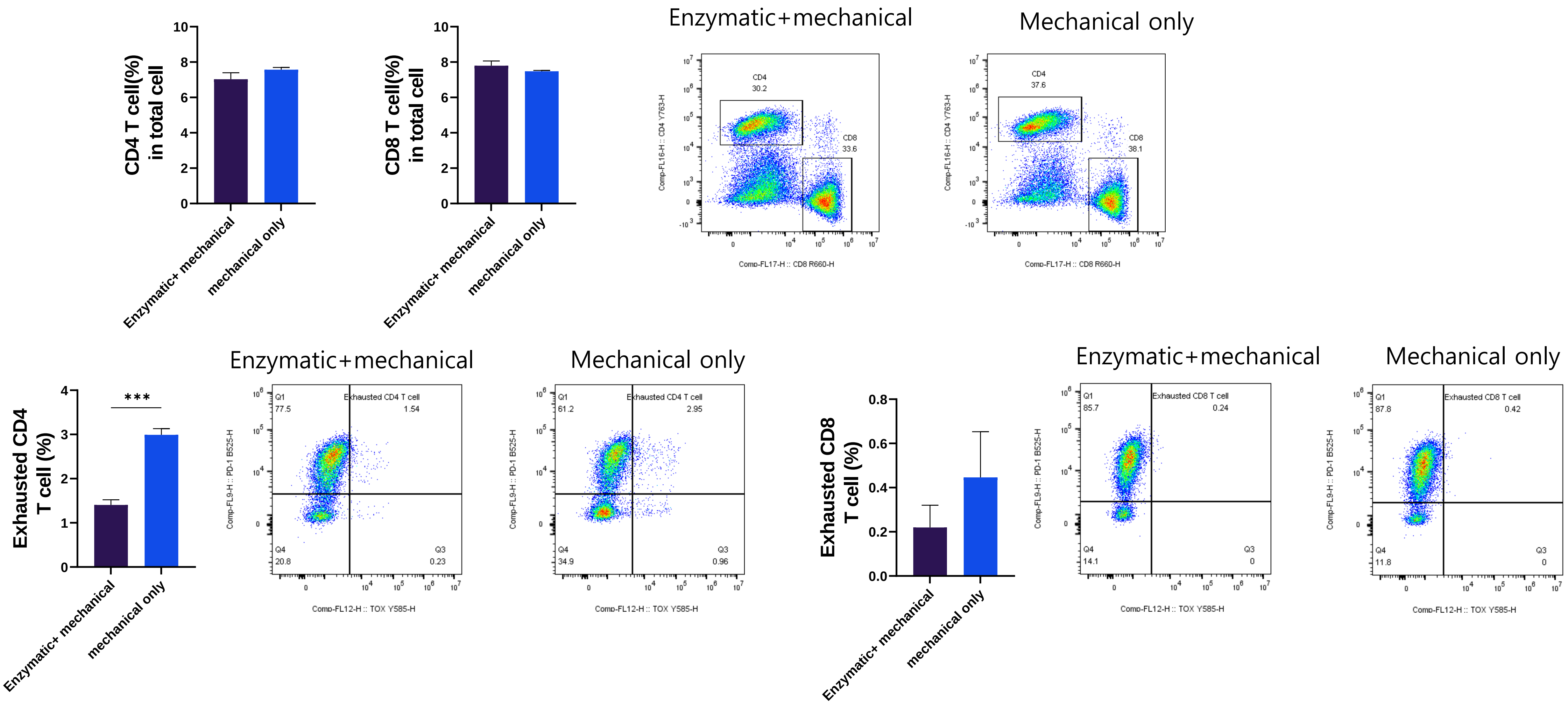

Supplementary figure 1

B. Comparisons of cytokine producing T cell populations depending on the lung disassociation methods

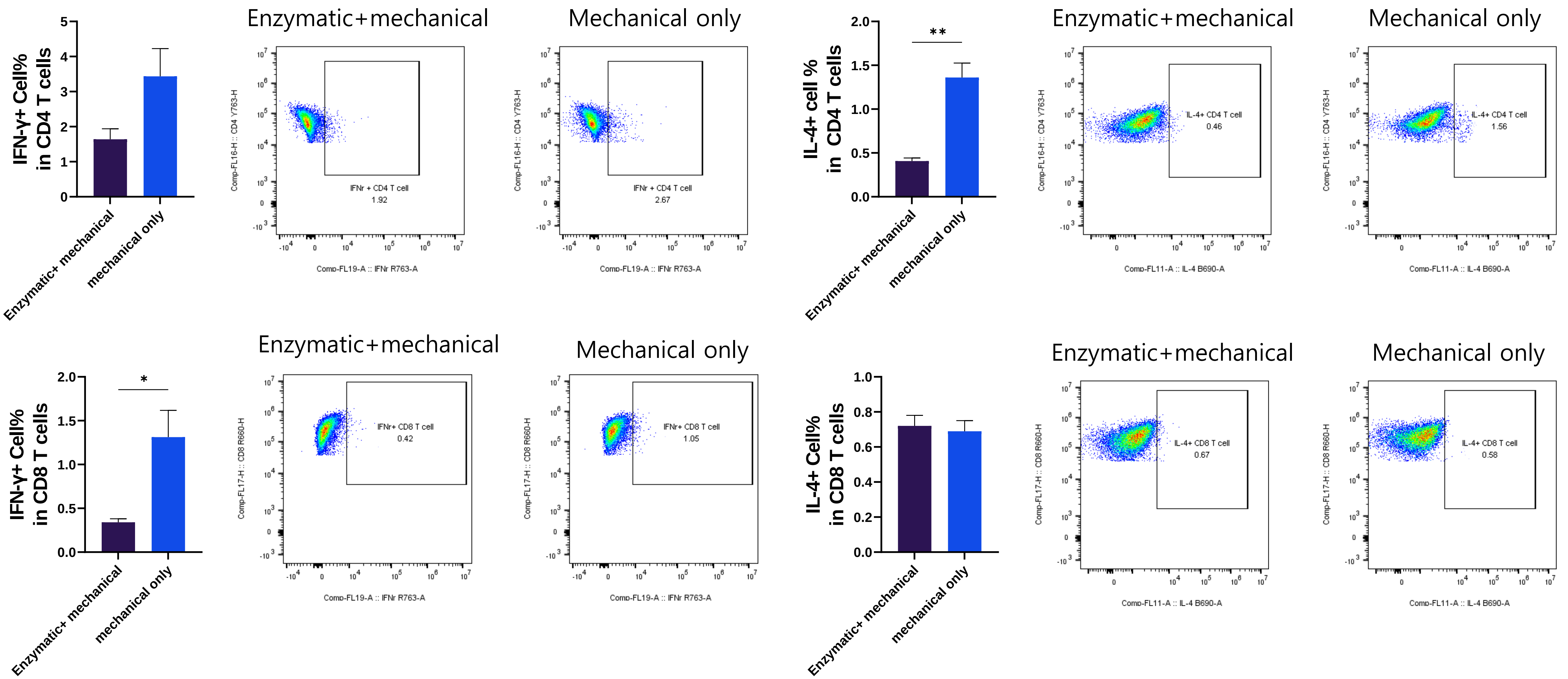

Supplementary figure 1

C. Comparisons of cell numbers of T cell populations depending on the lung disassociation methods

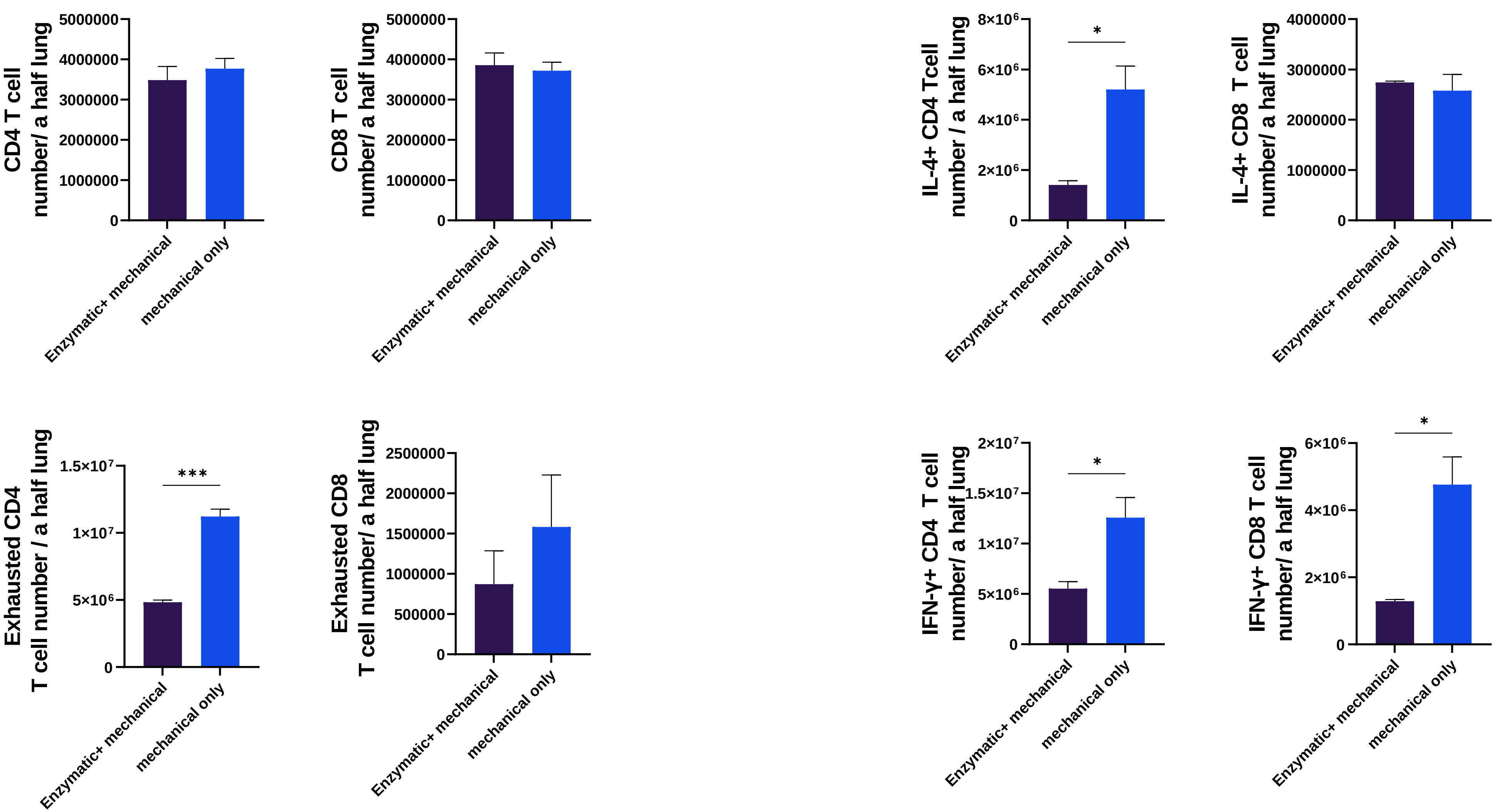

Supplementary figure 2

A. Inflammatory cell gating strategy

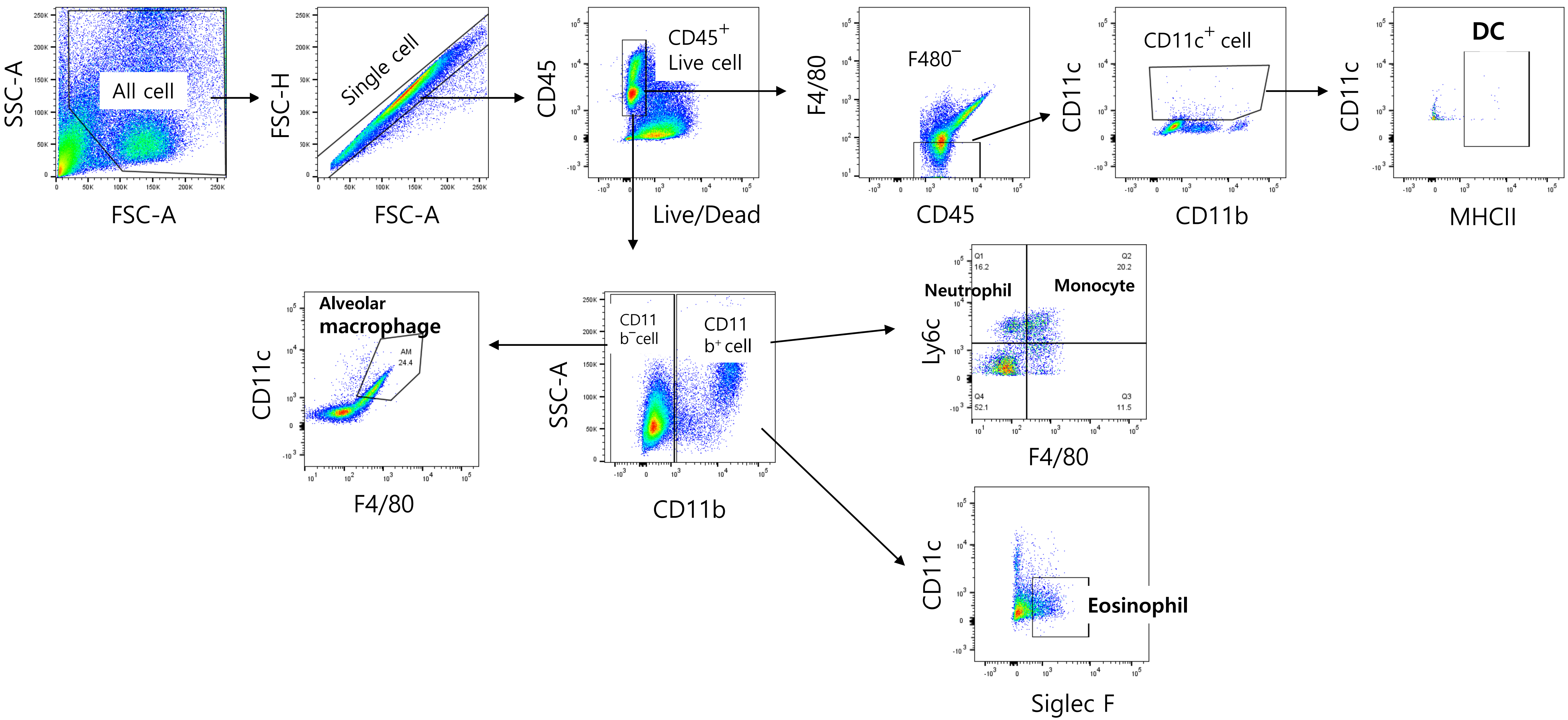

Supplementary figure 2

B. T cell IFN-γ, IL-4 intracellular cytokine staining gating strategy

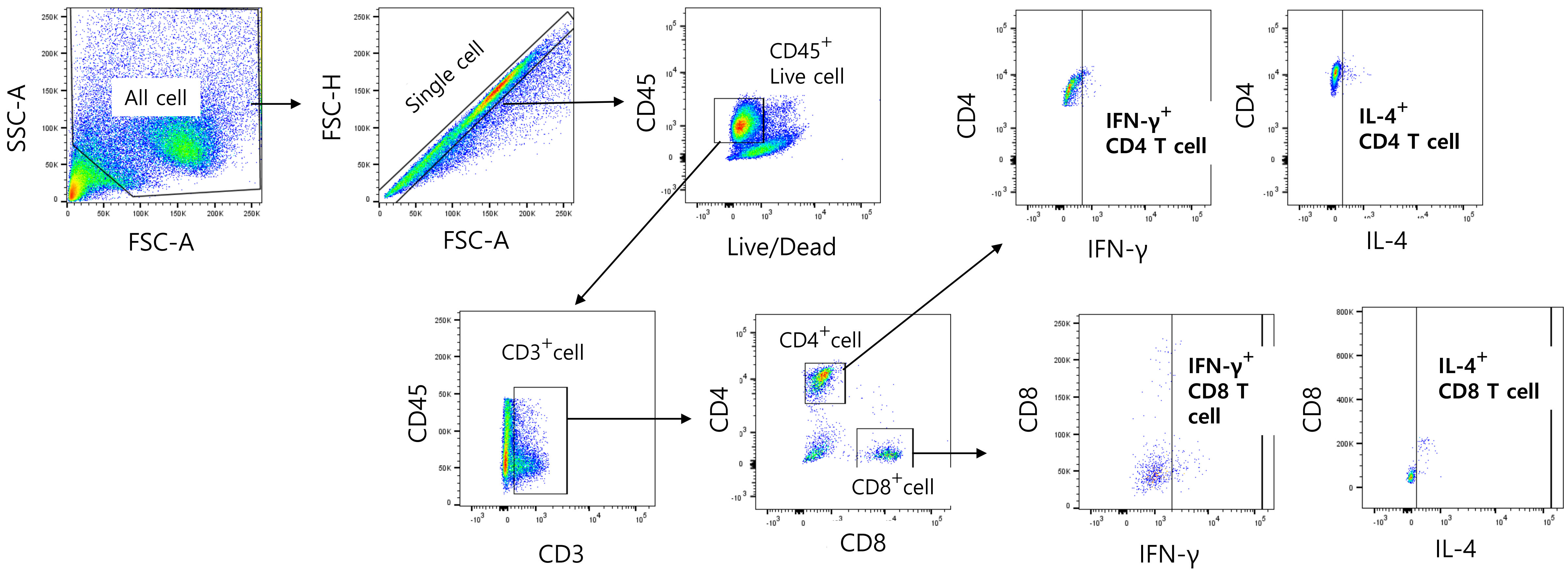

Supplementary figure 2

C. Exhausted T cell gating strategy

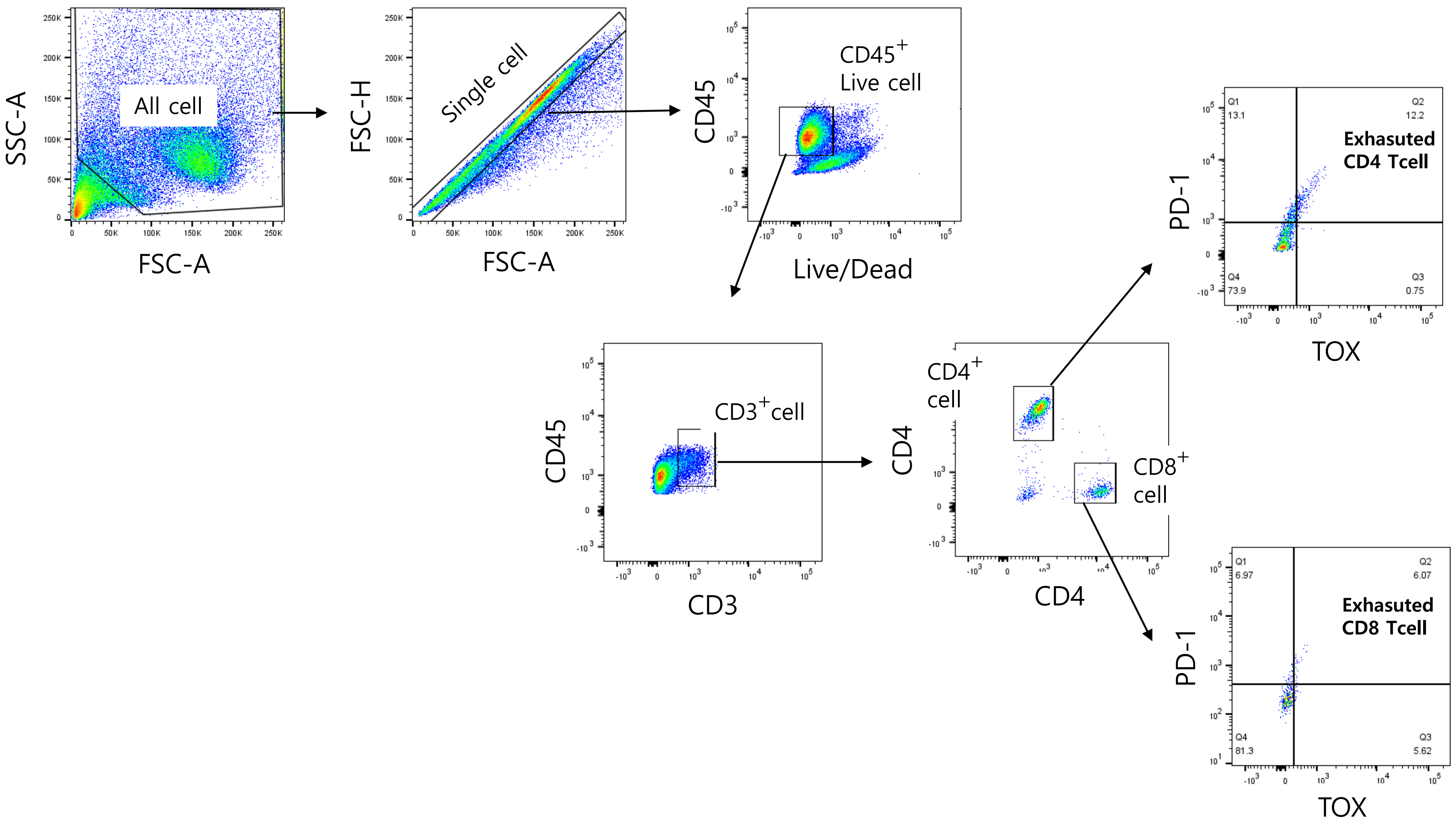

Supplementary figure 3

Lung inflammatory cell FSC-SSC Back gated result

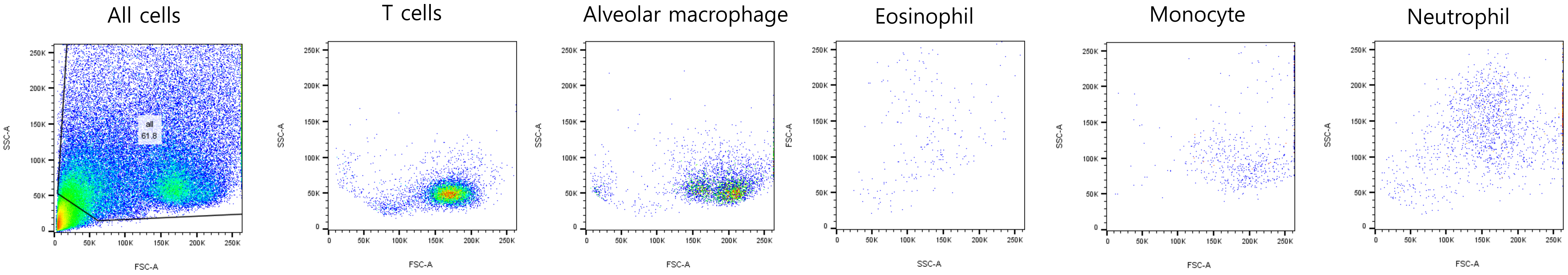

Supplementary figure 4

Inflammatory cell number in lung (A) and BALF (B)

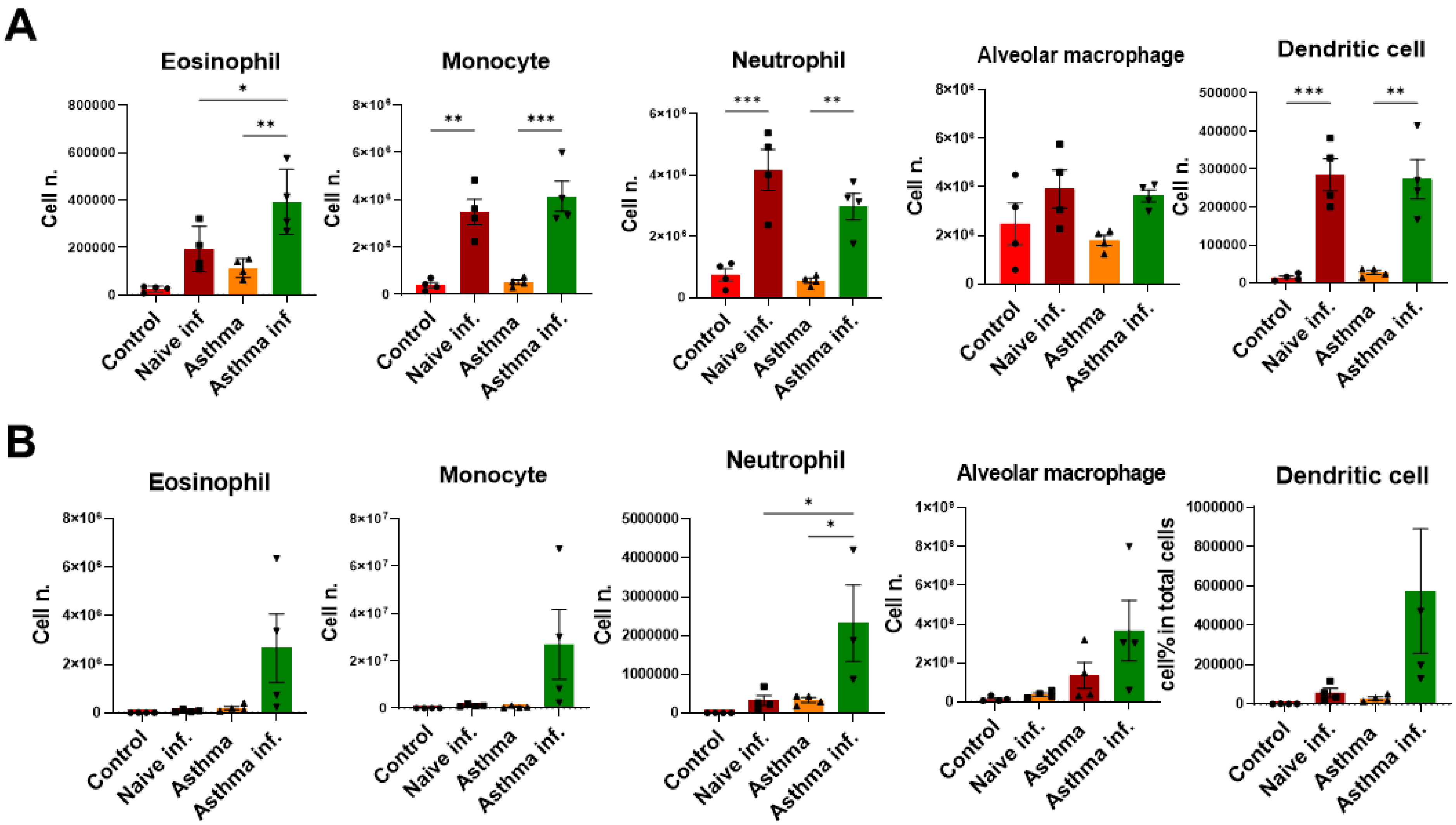

Supplement: Supplementary file 1 — Supplementary Material 1: Supplementary method Lung disassociation methods [file 12931_2023_2448_MOESM1_ESM.pdf]
